# Biosynthesis of Bacterial Cellulose by Extended Cultivation with Multiple Removal of BC Pellicles

**DOI:** 10.3390/polym13132118

**Published:** 2021-06-28

**Authors:** Ekaterina A. Skiba, Nadezhda A. Shavyrkina, Vera V. Budaeva, Anastasia E. Sitnikova, Anna A. Korchagina, Nikolay V. Bychin, Evgenia K. Gladysheva, Igor N. Pavlov, Andrey N. Zharikov, Vladimir G. Lubyansky, Elena N. Semyonova, Gennady V. Sakovich

**Affiliations:** 1 Institute for Problems of Chemical and Energetic Technologies, Siberian Branch of the Russian Academy of Sciences (IPCET SB RAS), 659322 Biysk, Altai Krai, Russia; eas08988@mail.ru (E.A.S.); shavyrkina.na@bti.secna.ru (N.A.S.); info@bti.secna.ru (A.E.S.); yakusheva89_21.ru@mail.ru (A.A.K.); labmineral@mail.ru (N.V.B.); evg-gladysheva@yandex.ru (E.K.G.); pawlow-in@mail.ru (I.N.P.); admin@ipcet.ru (G.V.S.); 2 Biysk Technological Institute, Polzunov Altai State Technical University, 659305 Biysk, Altai Krai, Russia; 3 Chair of Neymark Departmental Surgery and Hospital Surgery, Altai State Medical University, 656038 Barnaul, Altai Krai, Russia; zhar67@mail.ru (A.N.Z.); lvg51@mail.ru (V.G.L.); 4 Anatomic Pathology Department, Altai Krai Clinical Hospital, 656024 Barnaul, Altai Krai, Russia; translator@ipcet.ru

**Keywords:** bacterial cellulose, extended cultivation, pellicle removal, hernioplasty

## Abstract

Extended cultivation with multiple removal of BC pellicles is proposed herein as a new biosynthetic process for bacterial cellulose (BC). This method enhances the BC surface area by 5–11 times per unit volume of the growth medium, improving the economic efficiency of biosynthesis. The resultant BC gel-films were thin, transparent, and congruent. The degree of polymerization (DP) and elastic modulus (EM) depended on the number of BC pellicle removals, vessel shape, and volume. The quality of BC from removals II–III to VII was better than from removal I. The process scale-up of 1:40 by volume increased DP by 1.5 times and EM by 5 times. A fact was established that the symbiotic *Medusomyces gisevii* Sa-12 was adaptable to exhausted growth medium: the medium was able to biosynthesize BC for 60 days, while glucose ran low at 24 days. On extended cultivation, DP and EM were found to decline by 39–64% and 57–65%, respectively. The BC gel-films obtained upon removals I–VI were successfully trialed in experimental tension-free hernioplasty.

## 1. Introduction

The chemical purity and unique architecture of bacterial cellulose (BC) provide BC with properties such as high degree of polymerization, high crystallinity, mechanical strength, elasticity and shapeability, gas and liquid permeability, hydrophilicity, high water-holding capacity, nanoscale dimension, and nanoporosity [1,2]. The combination of these properties made BC an extremely attractive material for application both in conventional (pulp and paper, food, and chemical industries) and in innovative sectors of the global economy (biotechnological industry, biomedicine, electronics, and composite materials fabrication) [1,2,3,4,5,6]. Biomedicine has a special place where the successful use of BC is governed by exclusive properties of BC, such as biocompatibility, biodegradation, cell adhesion and proliferation capabilities, congruence, and transparency. The main applications of BC in biomedicine include wound and burns dressings, target drug delivery, scaffold for cell and tissue culture, and grafts (bone, cartilage, connective, cardiovascular, brain tunic, contact lens, vocal cords, dental prostheses, tympanic membrane, pinna, urinary bladder, etc.) [7,8,9,10,11,12,13]. That said, not only native BC but also modified BC, for example after physical treatment (stretching to make a uniaxially oriented film) [14] or acid treatment (sulfuric or acetic) for subsequent 3D printing [15], and BC-based composites are used.

Such a broad demand for BC is a powerful driving force for further research. Studies on synthesis methods of BC can be divided into three domains: (1) finding and genetic construction of new, highly productive microbial producers [1,9,16]; (2) development of new technological approaches to cultivation [4]; and (3) search for cheap alternative feedstocks [17,18,19,20]. The synthesis methods of BC determine BC properties which, in turn, determine its applications [2,10,21,22,23].

The cultivation methods of BC are classified into three types: static culture, dynamic culture, and bioreactor culture. The development of new culture techniques has pursued the aims to intensify the biosynthesis and attempt to design scalable reactors. However, despite the achievements made in dynamic and bioreactor culture techniques, and despite newly found applications of BC produced by these techniques, the loss of BC quality and yield is always noted in these methods. The static culture is a relatively simple method requiring no high-power inputs and therefore remains the most common process for BC. Besides, BC synthesized by the static culture represents a gel-film with excellent structure and properties [1,4,10].

The static culture method is well studied. The biosynthesis phases of BC have been established to correlate directly with cell growth phases, and the maximum buildup of BC is observed at a maximum cell count [16,24]. Mathematical models of cell growth, substrate/oxygen intake and BC biosynthesis have been designed [25]. The BC yield subject to the shape and material of the vessel in which static culture is performed has been studied [26]. Assumptions have been made about the mechanisms and phases of BC formation [14,27,28].

The thickening of cellulosic gel-films is known to occur due to the newly formed cellulose layers at the film-air interface [29]; this way, old pellicles sink down, while the new ones are formed on the surface of the growth medium, with deficient aeration in the pellicle stratum slowing down the biosynthesis of BC. Concurrently, there takes place nutrient depletion and metabolic by-product accumulation in the medium, that is, the biosynthesis of BC by static culture has classical limitations typical of any biotechnological process. The biosynthesis of BC can be intensified and extended by replenishing the nutrient medium [30,31] or by removal of BC pellicles. However, we have not found studies on repeated BC removal.

Therefore, we advanced a hypothesis that thin BC gel-films could repeatedly be taken out of the surface of the culture medium having an initial substrate loading that is optimum for BC biosynthesis (it is usually 20 g/L) [1]. It is well-known that the longer the biosynthesis, the more intergrown layers of gel-films are formed and the higher the overall thickness of the resultant gel-film [27,29]. However, if one sets an objective to produce thin gel-films, it is obvious that most of the substrate will remain unused. The initial substrate loading could be lowered, but then, the biosynthesis rate of BC would slow down [32,33]. Besides the substrate limitation [33], this is also explained by the low cell concentration in the poor medium. BC synthesis is known to be stimulated when the cell concentration rises and the cells reach a quorum [16,34].

Such gel-films with a greater surface area are demanded in prosthetic hernia repair of the anterior abdominal wall. The thin gel-films are congruent to warrant a good conformability to the internal organs, and are transparent to ease the work of a surgeon [13].

The present study aimed to explore the biosynthesis of BC by an extended cultivation method with multiple removal of BC pellicles where glucose is added once. The resultant gel-films were tested in prosthetic tension-free hernioplasty.

## 2. Materials and Methods

### 2.1. Microorganism and Inoculum Preparation

Symbiotic *Medusomyces gisevii* Sa-12 was acquired from the Scientific Center ‘Kurchatov Institute’―Research Institute for Genetics and Selection of Industrial Microorganisms (Russia). The symbiotic culture is composed of 15–30 yeast genera and 8–10 acetobacterium genera, also known as Kombucha. The species composition of the culture is detailed elsewhere [35,36,37].

The inoculum was cultured under static conditions at 27 ± 0.2 °C in a TC-80 hot-air thermostat (Russia) for 7 days. The active acidity was not adjusted during cultivation, which is optimum for most BC-producing microorganisms [1,17,20,32].

### 2.2. Preparation of Nutrient Media

To maintain the vital activity of *Medusomyces gisevii* and perform biosynthesis of BC, we employed a semisynthetic nutrient medium consisting of glucose (OOO Promsintez, CAS No. 5996-10-1, Chapayevsk City, Russia) and black tea extract. This medium is classical for the microbial producer used [38]. The medium was prepared as follows: a weighed portion of dry black tea (10 g dry black tea per 1 L nutrient medium, which is equivalent to 3.2 g/L black-tea extractives in the medium) was covered with boiling water and 20 g/L glucose was added thereto. The extraction vessel was then capped and the contents were held at 94–96 °C for 15 min, followed by filtration via a metal sieve. The nutrient broth was not sterilized, so the cultivation was run under non-sterile conditions.

### 2.3. Fermentation

Biosynthesis of BC was carried out under static conditions at 27 °C in a Binder-400 climate chamber (Berlin, Germany). The active acidity was not adjusted during cultivation. BC was biosynthesized in three vessels whose details are summarized in Table 1. The options differed in geometric dimensions and material the vessels were made of. Options 2 and 3 represent a scaled version of vessel 1, the scale-up ratio by volume being 1:40.

**Figure 1:**
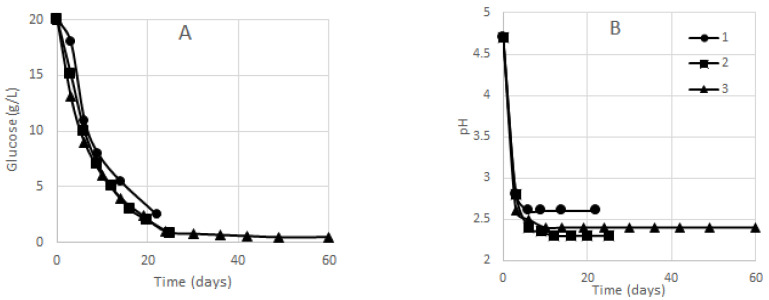
Time courses of (**A**) glucose concentration and (**B**) active acidity during biosynthesis of BC in vessels 1–3. The half-width of confidence interval was ±0.2 g/L for glucose concentration and ±0.1 for pH.

**Figure 2:**
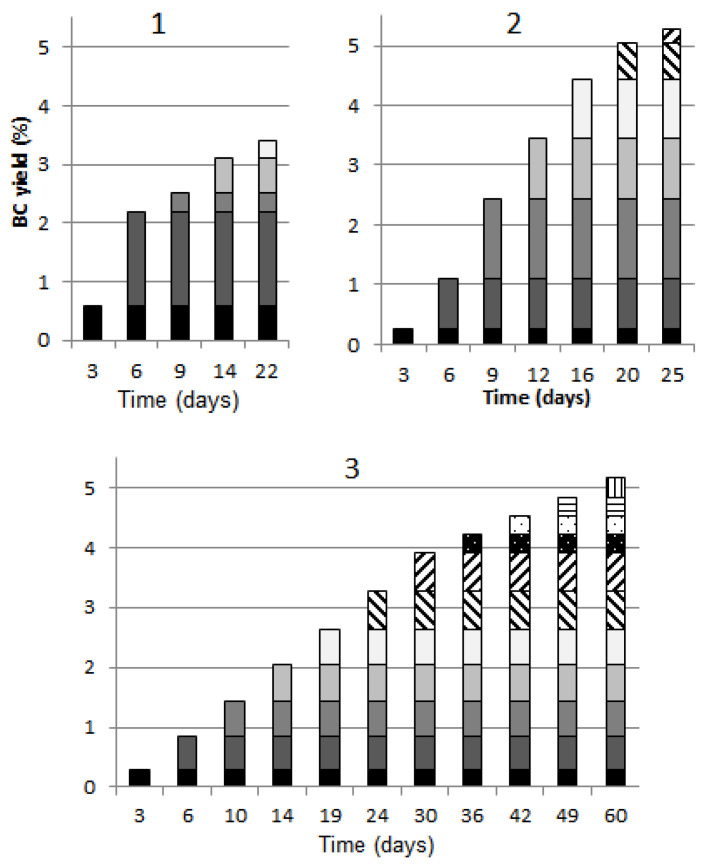
BC yield during biosynthesis in vessels 1–3. The BC yield upon each pellicle removal is marked with different color or hatching in the diagrams. The half-width of confidence interval for BC yield was ±0.1%.

**Figure 3:**
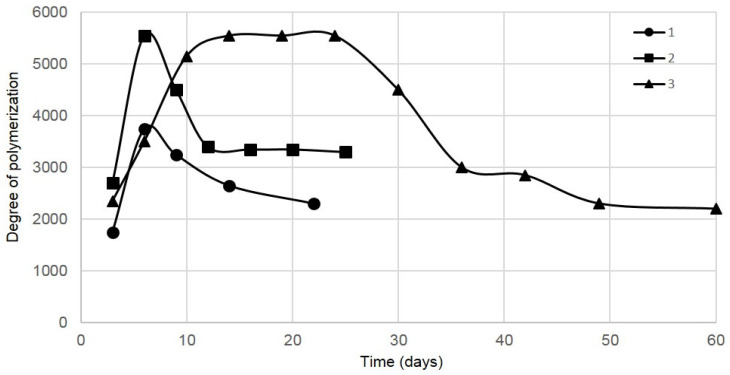
Degree of polymerization of BC samples obtained by extended culture in vessels 1–3. The half-width of confidence interval for degree of polymerization was ±100.

**Figure 4:**
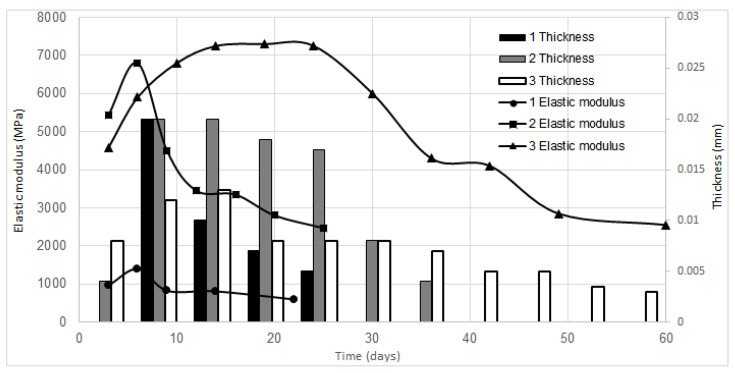
Thickness and elastic modulus of BC samples resulted from extended cultivation in vessels 1–3. The half-width of confidence interval was ±50 MPa for elastic modulus and ±0.0005 mm for BC thickness.

The time point to take out BC pellicles was determined visually in each option when a thin but dense gel-film was generated on the surface of the culture medium; the details are given in Table 2. Glucose was added once to the growth medium, and no more glucose was injected following removals of BC pellicles. Each experiment was done in duplicate. The statistical analysis was done by the common methods. For all the parameters given in Figures 1–4, the half-width of the confidence interval was identified at a significance level of 0.05.

### 2.4. Purification of BC

To avoid the alteration of the BC structure, BC was washed by the passive method at 20 °C [2]. The samples were washed with a 2% NaOH solution (CAS No. 1310-73-2, ОАО Kaustik, Volgograd, Russia) for 48 h and then with water until neutral pH, followed by decationation with HCl (AO LenReaktiv, CAS No. 7647-01-0, Saint-Petersburg, Russia) at pH 3 for 24 h, and again with water until neutral pH, as reported [32].

### 2.5. Calculation of BC Yield

The yield of BC was calculated by the equation:

$$\eta_{BC=\frac{m}{C_{G}\times V\times 0.9}\times 100\%}$$

where *η_BC_* is the *BC* yield (%); *m* is the weight of the oven-dried matter (g); *C_G_* is the initial glucose concentration in the medium (g/L); *V* is the nutrient medium volume (L); and 0.90 is the coefficient attributed to the water molecule detachment from glucose monomeric units during BC biosynthesis. A similar calculation was used in the other studies [39,40]. The calculation admits that the BC moisture is 99.0%, which has been established many times by us through gravimetric analysis after freeze-drying. It is consistent well with the literature data [27,41].

The weight of BC gel-films resulting from each subsequent removal of BC pellicle was summated, but the yield was always calculated on a basis of initial glucose concentration in the medium.

### 2.6. Analytical Techniques

The active acidity level was monitored by an I-160 MI ion meter (OOO Izmeritelnaya Tekhnika, Moscow, Russia). The glucose concentration was quantified by a spectrophotometer (United Products & Instruments, Dayton, NJ, USA) using 3,5-dinitrosalicylic acid (Panreac, CAS No. 609-99-4, Barcelona, Spain), as per the reported procedure [42]. The degree of polymerization was measured by a viscometer using cadoxene (ethylenediamine, AO LenReaktiv, CAS No. 107-15-3, Saint-Petersburg, Russia; cadmium oxide, AO LenReaktiv, CAS No. 1306-19-0, Saint-Petersburg, Russia), as described [43]. The thickness of BC samples was measured by an ICh-10 I-class dial Indicator thickness gauge (Kirov factory ‘Krasny Instrumentalshchik’, Kirov, Russia). The strength of BC was determined on a Shimadzu TMA-60 thermomechanical analyzer (Tokyo, Japan): the test sample was stretched at a rate of 0.5 g min^−1^ from 0.0 g to a maximum load of 400.0 g until failure, the test temperature was 23.0 °C. The analyses were carried out using equipment provided by the Biysk Regional Center for Shared Use of Scientific Equipment of the SB RAS (IPCET SB RAS, Biysk, Russia).

### 2.7. Prosthetic Tension-Free Hernioplasty

BC gel-films obtained in vessel 3 (removals I–VI) were tested as model hernia grafts in experiments on six dogs. BC was autoclaved for sterilization at 120 °C for 30 min (a steam sterilizer, OAO Tyumen Factory of Medical Equipment and Instruments, Russia). The animal experiments were approved by the local Ethics Committee of the Altai State Medical University of the Ministry of Health of the Russian Federation and performed in compliance with the international standards on animal care, animal husbandry, and humane treatment (European Convention for the Protection of Vertebrate Animals used for Experimental and Other Scientific Purposes, Strasbourg, 1986). Tension-free hernioplasty was simulated under general anesthesia in a sterile operating room by creating a total defect of the anterior abdominal wall and repairing it with a thin patch of the BC gel-film.

Unlike the previously reported results on using BC as a graft for prosthetic hernioplasty [13], here we elected to use gel-films with a minimum thickness to repair a large surface area of the hernia defect (total laparotomy). The minimum thickness of BC warrants its congruence with respect to the underlying tissues, a low tissue reaction to the material, as well as lightness and mobility within the abdominal wall. Increasing the surface area of BC samples is governed by the prospect of using such gel-films in the case of complicated postoperative and recurrent ventral hernias (20%) whose surgical treatment requires larger sizes of the material.

Sixty days following the placement of BC, the materials were harvested in the postoperative period and were visually evaluated for their location within the anterior abdominal tissues as well as for the presence of suppurative complications and BC adhesions to the subjacent bowel loops, followed by histologic examination of the tissue response to the graft.

## 3. Results and Discussion

### 3.1. The Number of BC Pellicle Removals

Table 2 summarizes experimental data on the number of pellicle removals done during the biosynthesis of BC in different vessels.

In the biosynthesis of BC in small vessel 1, we could take out BC pellicles five times, afterwards, the experiment was discontinued because of the depleted nutrient medium due to its small volume (only 0.2 L). The total biosynthesis time in vessel 1 was 22 days.

The highest number of BC pellicle removals (11 times) was achieved in vessel 3 having a tapered sphere shape, owing to which the biosynthesis had proceeded under limited aeration conditions, while the water evaporation intensity was the lowest, and the total biosynthesis time was the longest, 60 days. That is, the number of BC pellicle removals was increased by a factor of 2.2 and the duration extended by 2.7 times compared to vessel 1. Experiment 3 was discontinued because of nutrient depletion, as evidenced by Figure 1. The residual liquid weighed 784 g.

Cylinder-shaped vessel 2 holds an intermediate position: 7 BC pellicles were taken out, with a total biosynthesis time of 25 days. That is, the number of BC pellicle removals was increased by a factor of 1.4 and the duration extended by 1.1 times compared to vessel 1. The experiment was finished due to nutrient depletion. The residual liquid weighed 1720 g. In configurations 2 and 3, the residual liquids represented a slimy mass that was heterogeneous over the entire volume, and no gel-film was any longer formed on its surface upon further cultivation. According to Yassine et al. [28], this mass can be viewed as sparse cellulose, as opposed to the gel-film which the authors characterize as compact cellulose.

### 3.2. Glucose Concentration and BC Yield

The time courses of glucose concentration and active acidity during the biosynthesis of BC in vessels 1–3 are displayed in Figure 1. Representative curves were obtained, matching those reported in the literature for static culture performed without removal of BC pellicles [32,44,45].

The BC yield upon cultivation in vessels 1–3 is shown in Figure 2 where the BC yield is marked with different color or hatching for each BC pellicle removal. It is seen in the diagram that the highest yields were obtained upon BC pellicle removal II in vessel 1 and upon BC pellicle removal III in cylinder-shaped vessel 2, while almost equal yields were obtained in the spherical vessel upon removals II–VII. This can be explained by the shape of the vessels. When the surface area equals the vessel neck area, the gas and liquid exchange on the medium surface goes faster; hence, the liquid evaporates faster and biosynthesis of BC gel-films, whose microbial producer is a strict anaerobe, proceeds more intensively.

The low BC yield obtained in vessel 1 (3.3%) is associated with a rapid depletion of the nutrient broth, which is also due to evaporation of the culture medium whose initial layer height was only 3.6 cm. It follows from Figure 1 that glucose was not completely exhausted at the point the experiment was discontinued (the experiment was discontinued due to the exhausted liquid). The BC yields in cylinder-shaped vessel 2 and spherical vessel 3 were almost the same: 5.2% and 5.1%, respectively. The tapered-sphere shape slowed down the BC biosynthesis intensity; therefore, the biosynthesis time in vessel 3 increased by 2.4 times compared to cylinder-shape vessel 2 (60 days versus 25 days), though BC yields were equal. The biosynthesis of BC in vessel 3 having a tapered-sphere shape is of scientific interest and allows the maximum number of BC pellicles to be obtained per unit medium (11 versus 7 in cylinder-shaped vessel 2).

At 24 days, the glucose concentration in vessel 3 was below 1%; nonetheless, six more BC pellicles could further be taken out (removals VI–XI). This extremely intriguing phenomenon is explained by the metabolic features of acetobacteria. When glucose is converted into cellulose, the medium may simultaneously contain several cellulose precursors that are recordable in no way by spectrophotometry we use to measure the glucose concentration in the medium and are not visible as gel-films.

The second explanation of the observed phenomenon of the BC synthesis under glucose-free medium conditions is that symbiotic *Medusomyces gisevii* develops its own metabolic mechanism during its evolution whereby all the nutrients required for normal symbiotic functioning are synthesized by the symbiotic culture itself [37]. This symbiotic culture is able to switch from one substrate to another and utilize its own metabolic products as substrates. The classic example: acetobacteria contained in the symbiotic culture utilize ethanol the yeasts produce from glucose. Moreover, the symbiotic culture is capable of utilizing a variety of organic acids as well: acetic, gluconic, succinic, lactic and malic acids, and glycerol, which are its metabolic products [37]. Such a sophisticated but well-controlled metabolism in the symbiotic culture imparts this culture with a high adaptive potential; for instance, this culture is known to withstand heavy water and cold stress [46]. In the present study, we in fact demonstrate new adaptive capacities of the symbiotic culture, namely its ability to biosynthesize BC for 60 days under completely glucose-free conditions starting at 24 days. The fact that the symbiotic culture adapts to a depleted nutrient medium, and the biosynthesis of BC under these conditions is stable, suggests a high potential of *Medusomyces gisevii* for use in industry.

Since water in vessel 1 ran low faster than nutrients, the BC yields in vessels 2 and 3 are more adequate to compare not with vessel 1 but with the yield of 9% [32] obtained under steady-state conditions upon prolonged cultivation for 21 days (the same microbial producer and nutrient medium used). This way, the multiple removal of BC pellicles without scale-up gave a 2.7-fold decrease in BC yield. In the experiments on multiple removal of BC pellicles with a 1:40 scale-up by volume (vessels 2 and 3), the BC yield declined only by 1.8 times.

The significant fact is that, despite the reduced yield, the surface area of the resultant BCs increased manyfold (Table 3). The cultivation in vessel 1 without removal of BC pellicles could afford 65 cm^2^ BC. The cultivation in the same vessel with removal of BC pellicles increased the surface area of BC samples fivefold. At a 1:20 scale-up ratio by area (cylinder-shaped vessel 2), the surface area of BC samples increased by 142 times, while at a 1:15 scale-up ratio by area, but on condition that a tapered sphere-shaped container is used (vessel 3), the surface area increased by 160 times compared to single removal of BC pellicle in the small vessel. Namely, this is a combined effect of multiple BC removal and scale-up.

### 3.3. BC Properties

An interesting issue is the quality of BC samples obtained at different number of gel-film removals. Here we evaluated the quality by two parameters: degree of polymerization that is a universal characteristic of cellulose of any origin, and elastic modulus that characterizes the strength of samples, which is of practical importance.

Figure 3 shows the degree of polymerization (DP) of BC samples obtained by extended cultivation with multiple removal of BC pellicles.

The highest DP in vessel 1 was documented when the glucose consumption and the BC biosynthesis rate were maximum. This corresponded to pellicle removal II, afterwards the DP declined abruptly: by 39–40% by the end of biosynthesis (compared to its maximum value). In vessel 3 having a tapered sphere shape, the BC samples obtained by pellicle removals III–VI were found to have almost equal maximum DPs. Afterwards, the DP declined abruptly upon removals VII–XI: by 60% by the end of biosynthesis (compared to its maximum value).

The scale-up by volume enhanced the DP of BC samples. In vessels 2 and 3, the maximum DP was 1.5 times as great as that in small vessel 1 (3750 vs. 5550).

The phenomenon of the reduced DP by extended cultivation was described once in the literature. Bikales & Segal [47] explained it by destruction of BC; in contrast, we observed the production of BC rather than destruction. Shi et al. [48] made no assumptions of the nature of the observed phenomenon. In our previous study [24], we hypothesized that this could be explained physiologically: the cell population condition worsens over time, that is, the nutrients are running low and metabolic products are building up, therefore the DP of the synthesized BC declines as well. The present study has proved our earlier suggested hypothesis.

The thickness and elastic modulus of BC samples during the biosynthesis in vessels 1–3 are depicted in Figure 4. In all three biosynthesis options, it is quite natural that the higher the BC yield obtained from the corresponding BC removal, the thicker the resultant BC pellicle. The elastic modulus increased in direct proportion to the increase in pellicle thickness.

A very interesting relationship was established between elastic modulus and the number of BC pellicle removals and biosynthesis vessel shape. In vessels 1 and 2, the highest elastic modulus was documented when glucose uptake and BC biosynthesis rate were the highest. This corresponded to pellicle removal II afterwards the elastic modulus declined sharply by 57% for vessel 1 and 64% for vessel 2 compared to its maximum value. In vessel 3 having a tapered-sphere shape, BC samples obtained upon pellicle removals III–VI were observed to have nearly the same elastic modulus, afterwards the elastic modulus upon pellicle removals VII–XI declined sharply by 65% compared to its maximum value.

The elastic modulus dependence of the vessel volume can be considered paradoxical. The maximum thickness of the samples in vessels 1 and 2 was the same, 0.02 mm, but the elastic modulus of the BC samples produced in small vessel 1 (0.2-L nutrient medium) was 4.3 times as low as that of the BC samples obtained in vessel 2 (8-L nutrient medium): 1400 MPa versus 6050 MPa. That is, the scale-up by volume increased the elastic modulus of the BC samples. The highest elastic modulus of 7300 MPa was documented for BC samples derived in tapered sphere-shaped vessel 3, which is 5.2 higher than that of the samples obtained in vessel 1. That being said, the thickness of the samples produced in vessel 3 was 1.5 times as low as that of the samples obtained in vessels 1 and 2, i.e., 0.013 mm. This can be attributed to biosynthesis under conditions of limited aeration.

In our previous study, the cultivation without removal of BC pellicles (under the same other experimental conditions) afforded a BC sample having an elastic modulus of 933 MPa [32]. That is, the elastic modulus was 1.5 times higher in vessel 1, 7 times higher in vessel 2, and 7.8 times higher in vessel 3 than that without removal of BC pellicles (maxima are compared). It is difficult to compare these experimental results with the literature because such a cultivation has not been described before. The obtained elastic moduli range from 600 to 7300 MPa. The world literature reports both lower elastic moduli from 10 to 17 MPa [41] and higher elastic moduli from 15 to 138 GPa [1]. Molina-Ramírez et al. [49] obtained the opposite results by scaling up the nutrient medium volume from 90 mL to 4 L (1:44 scale-up ratio), i.e., the elastic modulus declined from 1150 to 569 MPa, that is, twofold.

The commonly used methods for characterization of BC include also SEM, FTIR and X-ray diffraction. The imaging of BC can identify the arrangement of the pellicle network and determine porosity, morphology and size of the fibers. Unlike any other cellulose types, the BC structure represents a disordered interlacement of microfibrils 25 to 200 nm wide [9]. As we showed previously for our microbial producer used, the average width of microfibrils was 30.6 nm (a single pellicle removal from a 100 mL nutrient medium) [32].

FTIR can prove that the test substance is actually cellulose. For the microbial producer we used, that was demonstrated in our previous studies many times [19,32,39,50]. Besides, FTIR enables the measurements of the crystallinity index and the ratio of allomorph I α to I β in BC, more specifically these parameters allow the individual structure of BC to be identified, that is, how one type of BC differentiates from another BC type and what applications it may find. However, the most reliable method to determine the index of crystallinity and the I α to I β allomorph ratio in BC is X-ray diffraction which can be considered as a referee method [51,52].

In our previous paper [19], it was demonstrated that the variation in the nutrient medium composition and the inhibitors of BC biosynthesis contained therein did not alter the index of crystallinity and the I α to I β allomorph ratio in the BC test samples. The values were very high: the index of crystallinity was 86–94% and the allomorph I α content was 99–100%. This is surprising and contrary to most of the studies [1,20]. However, we are convinced that our results are valid and confirmed by our colleagues’ studies [50,53,54] in which the multimicrobial Kombucha culture was exposed to simulated Mars-like conditions in the low-Earth orbit. The microecosystem of the symbiotic culture showed a high survivability, and the BC structure did not show any significant changes in all kinds of tests. Thus, our colleagues have confirmed our inferences that the unchangeable BC structure is explained exactly by the properties of the microbial producer *Medusomyces gisevii*.

Nonetheless, a question remains unanswered in the present study as to how the multiple removal of BC pellicles influences the BC structure? What exactly will be happening upon depletion of nutrient medium and buildup of metabolic products? How the symbiotic *Medusomyces gisevii* will respond to these challenges in terms of preservation or variation of the BC structure because BC is a protective cage for a symbiotic community! How exactly do the BC micro- and nanoarchitectures, fiber size, and porosity change and hence the individual parameters such as index of crystallinity and the I α to I β allomorph ratio? Or the symbiotic culture is able to retain them? These are very interesting fundamental questions whose answers will help better understand both the BC synthesis mechanism and its influencing factors. In our further study, we hope to give full answers to these questions.

### 3.4. Outcomes of Tension-Free Hernioplasty

The implanted material and the living organism when contacted are known to be prone to a mutual interaction of negative nature in general [55]. Earlier performed numerous experimental and clinical studies demonstrated that aseptic inflammation developed following surgical intervention for inguinal hernia repair [56,57,58]. The neutrophilic stage of inflammation comes within the first post-surgery hours, with the lymphocytes from the vessels migrating towards the irritation source, surrounding it to form a leucocytic bank in 6–12 h. The resulting macrophages subsequently infiltrate the leucocytic bank, followed by phagocytosis of cell debris and proliferation of fibroblasts that are ones involved in the formation of collagen fibers [59]. Thus, the response to the foreign body with temporal development of aseptic inflammation and outgrowth of granulation tissue followed by its maturation into the fibrous tissue can be regarded as a normal reaction to the biocompatible material. However, the occurrence and severity of the inflammatory response depends not only on the graft properties, but also on the area of the surface being in contact with the recipient’s tissues [60].

**Figure 5:**
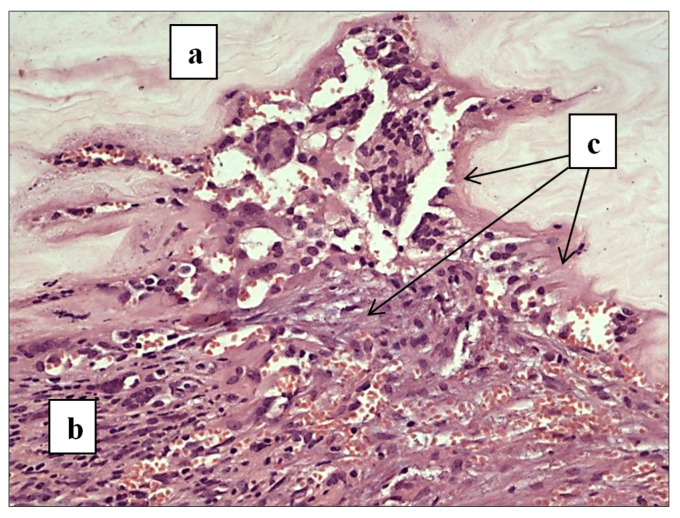
Histologic examination of BC gel-film (obtained in vessel 3, removal II) and anterior abdominal wall tissues at 60 days post-implantation: (**a**) BC gel-film, (**b**) anterior abdominal tissues, and (**c**) fixation boundary of BC gel-film with a histologic presentation of aseptic inflammation resolving and this site being replaced by collagenous structures. Hematoxylin and eosin staining. 20× zoom.

In our study, the macroscopic examination of the BC location in the anterior abdominal wall tissues demonstrated that the BC with a smooth and wet surface biointegrated into the anterior abdominal wall tissues, showing an initial (slight) borderline aseptic inflammation (Figure 5) and, finally, signs of mild formation of collagenous and vascular structures at the site of the hernia graft without rough scarring around the prosthetic device. The quality of the hernia graft was found not to be dependent on how many times BC gel-films were taken out of the culture medium surface.

There were no suppurative sequalae throughout the entire postoperative period. It is important to note a negligible quantity of BC adhesions to the underlying bowel loops when BC was placed intraperitoneally, which can be explained by the smooth surface of the BC gel-film, mild initial aseptic inflammation with respect to BC, and the absence of fibrous downgrowth. When a polypropylene mesh is used, the resulting dense connective-scar tissue downgrows into the mesh cells, making the mesh poorly movable and “heavy”. The low adhesion of BC to the internal organs of the abdomen is crucial in patients having large postoperative ventral hernias with a tissue-deficient anterior abdominal wall and opens up prospects for using BC in surgery and veterinary medicine in the future.

Figure 5 illustrates histologic examination of the BC patch and anterior abdominal wall tissues 60 days post-implantation. At the interface of BC with the anterior abdominal wall tissues, one can see the formation of the boundary of the material fixation, with elements of mild aseptic inflammation.

Thus, it is the thin BC gel-films that warrant the formation of a lightweight hernia graft through its rapid and gentle saturation with fibroblasts, macrophages and intercellular substance, without emergence of rough scar tissues and massive adhesion to the underlying organs of the abdomen. This abates the rigidity (ankylosis) of the inner connective-tissue scar observed with a polypropylene mesh and can prospectively improve the quality of life in postoperative patients.

## 4. Conclusions

Here, we introduced an extended cultivation method with multiple removal of BC pellicles as a new strategy for the biosynthesis of bacterial cellulose (BC). This process considerably increases the surface area of BC gel-films per unit volume of the nutrient medium by a factor of 5–11, depending on the vessel shape and volume.

Besides the high surface areas of BC gel-films obtained by pellicle removals II and III, the degree of polymerization and elastic modulus also increased as compared to the single-time removal of a BC pellicle. Moreover, the scale-up of 1:40 by volume raised the degree of polymerization by 3.4 times (5550) and the elastic modulus by 4.9 times (to 6800 MPa) compared to the small vessel. When the sphere-shaped vessel, having a surface area 2.8 times as great as its neck area, was used, the maxima of the degree of polymerization and elastic modulus were documented upon pellicle removals III–VI, with the elastic modulus increasing even more by 5.32 times (to 7300 MPa).

It was established that the symbiotic *Medusomyces gisevii* Sa-12 is adaptable to the depleted nutrient medium: the culture can produce BC for 60 days in a completely glucose-free medium starting at 24 days. However, the extended cultivation decreased the elastic modulus (by 57–65%) and degree of polymerization (by 39–64%) of BC samples compared to their maxima, which is due to depleted nutrients and accumulated metabolic products.

The resulting pellicles are thin, transparent, and congruent and are meant for medical use. The experiment on dogs demonstrated that the outcome of the test prosthetic tension-free hernioplasty of the anterior abdominal wall did not depend on how many times the BC gel-films were taken out (removals I–VI) of the growth medium surface. The low tissue reaction and low adhesive properties were found to ensure successful hernia repair in the experiment using thin BC gel-films of larger size. This opens up prospects for hernioplasty using BC in surgery and veterinary medicine in the future.

## Figures and Tables

**Table 1:** Specifications of vessels used for bacterial cellulose (BC) biosynthesis, and culture medium volume.

Option	1	2	3
Vessel material	Food-grade polypropylene	Enamelled iron	Glass
Vessel shape	Rectangular parallelepiped	Cylinder	Tapered sphere
Geometric dimensions, cm	Length 9.0 Width 6.4 Height 4.8	Radius 20.5 Height 35	Radius 17.4 Neck radius 10.4
Ratio of growth medium area to vessel neck area	1:1	1:1	2.8:1
Growth medium surface area *S*, cm^2^	65	1320	946
Scale-up ratio by area	-	1:20	1:15
Vessel volume, L	0.25	45.0	17.3
Growth medium volume, L	0.2	8.0	8.0
Scale-up ratio by volume	-	1:40	1:40
Growth medium layer height *h*, cm	3.6	6.1	from 0 to 12.5
Ratio of *S/h*, cm	18	218	from 0 to 76

**Table 2:** Experimental data on the number of BC pellicle removals during biosynthesis of BC in different vessels.

Gel-Film Removals	I	II	III	IV	V	VI	VII	VIII	IX	X	XI
**Biosynthesis in small vessel 1**											
Biosynthesis time for a given BC sample (days)	3	3	3	5	8	–	–	–	–	–	–
Total biosynthesis time (days)	3	6	9	14	22	–	–	–	–	–	–
**Biosynthesis in cylinder-shaped vessel 2**											
Biosynthesis time for a given BC sample (days)	3	3	3	3	4	4	5	–	–	–	–
Total biosynthesis time (days)	3	6	9	12	16	20	25	–	–	–	–
**Biosynthesis in tapered sphere-shaped vessel 3**											
Biosynthesis time for a given BC sample (days)	3	3	4	4	5	5	6	6	6	7	11
Total biosynthesis time (days)	3	6	10	14	19	24	30	36	42	49	60

**Table 3:** Overall surface area of BC samples obtained in different vessels.

Vessel	1	2	3
Surface area of growth medium *S*, cm^2^	65	1320	946
Number of gel-films	5	7	11
Overall surface area of BC samples, cm^2^	325	9240	10,406

## Data Availability

The data that support the findings of this study are available from the corresponding author upon reasonable request.
